# Ammonia Emission Sources Characteristics and Emission Factor Uncertainty at Liquefied Natural Gas Power Plants

**DOI:** 10.3390/ijerph17113758

**Published:** 2020-05-26

**Authors:** Seongmin Kang, Seong-Dong Kim, Eui-Chan Jeon

**Affiliations:** 1 Climate Change & Environment Research Center, Sejong University, Seoul 05006, Korea; smkang9804@gmail.com; 2 Cooperate Course for Climate Change, Sejong University, Seoul 05006, Korea; kevin24304@naver.com; 3 Department of Climate and Environment, Sejong University, Seoul 05006, Korea

**Keywords:** PM 2.5 secondary sources, SCR, LNG power plant, ammonia emission factor, uncertainty

## Abstract

This study developed the NH_3_ emission factor for Liquefied Natural Gas (LNG) power facilities in Korea by analyzing the emission characteristics from two LNG power plants using methods such as uncertainty analysis. Also, comparing the differences in NH_3_ emission levels between the developed emission factors, which reflect the characteristics in Korea, and the U.S. Environmental Protection Agency (EPA) values currently applied in Korea. The estimation showed that the NH_3_ emission factor for the LNG power plants was 0.0054 ton NH_3_/10^6^Nm^3^, which is approximately nine times less than the EPA NH_3_ emission factor of 0.051 ton NH_3_/10^6^Nm^3^ for LNG fuels of the industrial energy combustion sector currently applied in national statistics in Korea. The Selective Catalytic Reduction (SCR) emission factor for LNG power plants was 0.0010 ton NH_3_/10^6^Nm^3^, which is considerably lower than the EPA NH_3_ emission factor of 0.146 ton NH_3_/10^6^Nm^3^ currently applied in national statistics in Korea for the LNG fuels of the industrial process sector. This indicated the need for developing an emission factor that incorporates the unique characteristics in Korea. The uncertainty range of the LNG stack NH_3_ emission factor developed in this study was ±10.91% at a 95% confidence level, while that of the SCR NH_3_ emission factor was –10% to +20% at a 95% confidence level, indicating a slightly higher uncertainty range than the LNG stack. At present, quantitative analysis of air pollutants is difficult because numerical values of the uncertainty are not available. However, quantitative analysis might be possible using the methods applied in this study to estimate uncertainty.

## 1. Introduction

In 2018, the fine particulate matter concentration in Korea was 24 μg/m^3^, which is the second highest concentration after Chile when compared to other members of the Organization for Economic Co-operation and Development. This level was approximately two times higher than other advanced countries such as the UK, Japan, and France [1].

One reason for the increasing concentration of fine particulate matter may be the increase in secondary aerosols. The substances that are involved in the secondary generation of particulate matter include NH_3_, SOx, NOx, and volatile organic compounds [2,3,4,5]. To reduce air pollutants such as particulate matter, several policies have been implemented in Korea [6,7,8]. however, these policies only focus only on the management of NOx and SOx. There is insufficient research regarding the identification of the emission source or the application of the emission factor of NH_3_ in Korea.

In Korea, an inventory of air pollutants has been established, which is categorized into manufacturing industry combustion, energy industry combustion, nonindustry combustion, production process, off-road mobile pollution sources, agricultural sources, waste treatment sources, biological combustion, and other sources. Of the air pollutants, NH_3_ emission levels of Liquefied Natural Gas (LNG) power are the second highest, after bituminous coal power, in the energy industry combustion category. The 1994 U.S. Environmental Protection Agency (EPA) values were applied to the LNG power plant NH_3_ emission factor, which indicates the difficulty of incorporating the unique characteristics in Korea [9,10].

In Korea, the NH_3_ emission source in the production process category at a power plant also includes NH_3_ emissions due to selective catalytic reduction (SCR). Therefore, the purpose of this study was to analyze and assess NH_3_ emissions at LNG power facilities in Korea relevant to the emission factor formulation or uncertainty analysis. This study also examined the differences between the currently applied EPA values and emission factors in the U.S. and the application of emission factors in Korea to determine whether the differences are reflected with respect to NH_3_ emission levels in the two countries.

## 2. Methods

### 2.1. Selection of Objective Facilities

Since most LNG power plants in Korea are almost combined cycle power plants, the sampling target was for LNG combined cycle power plants, and it was difficult to obtain cooperation from the power plants, so only two sites could conduct research. This study collected a minimum of three NH_3_ samples from two LNG power plants to identify the NH_3_ emission source characteristics. The power generation capacity, daily average fuel consumption, daily average flow rate and number of samples from the power plants are presented in Table 1.

**Figure 1:**
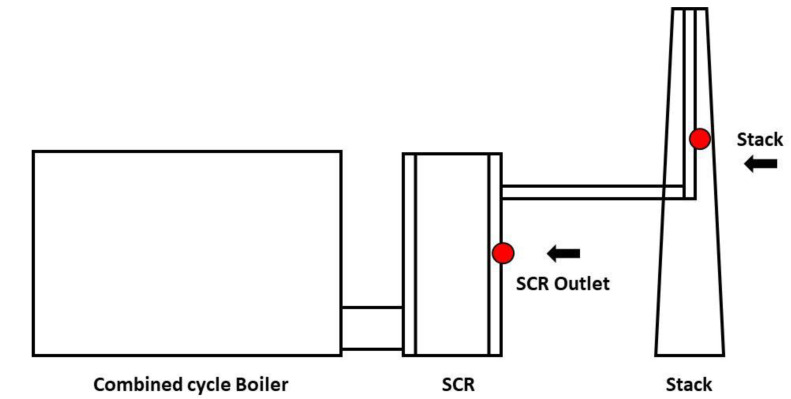
Schematic of the sampling point and air pollution prevention at Liquefied Natural Gas (LNG) power plants.

Additional NH_3_ sampling at one of the power plants was concurrently carried out at the SCR outlet to analyze the differences in NH_3_ emission sources, with the purpose of potential inventory improvement. Air pollution prevention facilities installed only SCRs, which are NOx reduction facilities, at both LNG plants A and B. The approximate sampling location and related schematic diagram are shown in Figure 1.

### 2.2. NH_3_ Analysis at LNG Power Plants

**Figure 2:**
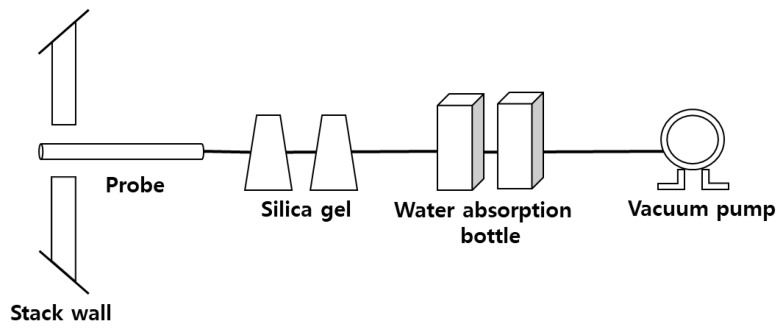
Schematic of the field setup for ammonia sampling at LNG power plant.

To measure the NH_3_ concentration at an LNG power plant, the indophenol method suggested in the odor and air process test methods was used. The indophenol method quantifies NH_3_ concentrations based on the absorbance of indophenols generated during the reaction with NH_4_^+^ ions in the analytic sample upon addition of sodium hypochlorite and phenol-sodium nitroprusside solutions. For NH_3_ sampling, an NH_3_ absorbent (50 mL boric acid solution) was placed in two 50 mL flasks and exhaust gas was added using a minipump at a rate of 4 L/min for 20 min, for a total of 80 L. To remove the moisture from the exhaust gas, a bottle containing silica gel was placed at the entrance of the NH_3_ sampling device. Figure 2 shows a schematic diagram of the NH_3_ sampling process. After NH_3_ collection, the outside diameter of the absorbent was measured at 640 nm using a spectrophotometer. NH_3_ sampling was performed at the power plant stack and the SCR outlet for power plant A and only at the stack for power plant B.

### 2.3. Development of NH_3_ Emission Factor

The NH_3_ emission factor formula is shown in Equation (1). The development of the NH_3_ factor utilized the emission-based emission factor development method, which is a method used for the development of the NH_3_ emission factor for bituminous coal power plants and coke of NH_3_ emission factor [11,12]. This method calculates emissions by multiplying the flow rate of a combustion facility by ammonia concentration and dividing it by fuel consumption to obtain a fuel consumption-based emission factor. In the case of ammonia concentration, the ammonia concentration on the basis of the measurement was calculated and the corresponding flow rate and fuel consumption were provided from the LNG power plant.

(1) $$EF_{NH_{3}}=\left[C_{NH_{3}}\times \frac{M_{w}}{V_{m}}\times Q_{day}\times 10^{-6}\right]/FC_{day}$$

where EF is emission factor (ton NH_3_/10^6^Nm^3^); $C_{NH_{3}}$ is NH_3_ concentration in exhaust gas (ppm, NH_3_ μmol/air mol); $M_{w}$ is molecular weight of NH_3_ (constant) = 17.031 (g/mol NH_3_); $V_{m}$ is one mole ideal gas volume in standardized condition (constant) = 22.4 (m^3^/air mol); $Q_{day}$ is daily accumulated flow rate (Sm^3^/day) (based on dry combustion gas); and $FC_{day}$ is daily fuel consumption (Nm^3^/day).

### 2.4. Uncertainty Analysis by Monte Carlo Simulation

**Figure 3:**
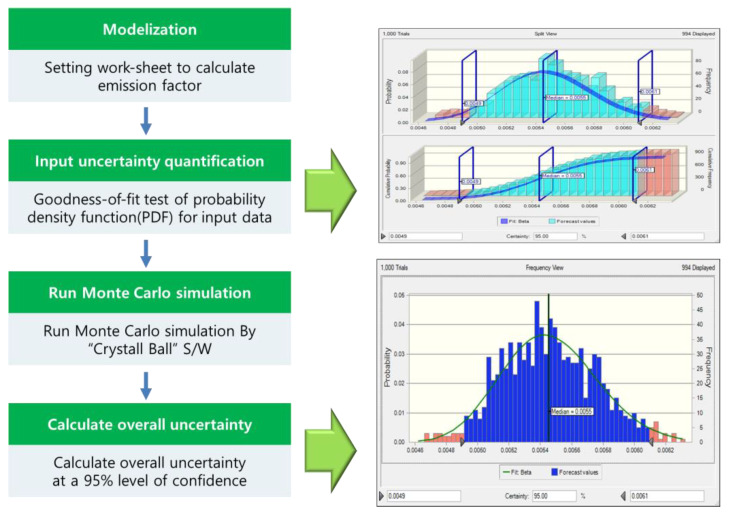
Process of the Monte Carlo Simulation for estimating the uncertainty of the emission factor.

The Monte Carlo simulation was utilized to estimate the uncertainty of the NH_3_ emission factor. This method evaluates the uncertainty by generating random numbers and assigning a probability density function (PDF) to each variable [13,14]. The Intergovernmental Panel on Climate Change (IPCC) recommends the Monte Carlo simulation as a Tier 2 method of estimating the uncertainty of greenhouse gas (GHG) emission factors. As shown in Figure 3, the analysis based on the Monte Carlo simulation involves four steps. The first step selects the appropriate model and composes the NH_3_ emission factor estimate work sheet. In the second step, the PDF conformance of the input variable required for development of the NH_3_ emission factor is tested. The significance level for the hypothesis testing was set to 5%. In addition, based on the conformance tests of the NH_3_ emission concentration, the PDF of the emission flow rate and fuel consumption (which are necessary to determine the NH_3_ emission factor) are estimated. The Monte Carlo simulation is performed in step three, where Crystal Ball is used for random sampling simulation. In step four, the uncertainty range is estimated based on the simulated results at a 95% confidence interval.

## 3. Results and Discussion

### 3.1. Characteristics of NH_3_ Emissions

The NH_3_ concentration results at power plants A and B are presented in Table 2. The mean NH_3_ concentration at power plant A was 0.05 ppm with a standard deviation of 0.03 ppm. The mean NH_3_ concentration at the SCR outlet at power plant A was 0.04 ppm with a standard deviation of 0.02 ppm. The mean NH_3_ concentration at power plant B was 0.18 ppm with a standard deviation of 0.17 ppm, which is three times higher than that of power plant A. This is attributed to additional NH_3_ utilized in the SCR process at power plant A, which reduces the NO_x_ concentration; however, the reduction of the unreacted NH_3_ leads to its emission through the stack [11,12,15]. Therefore, when the NO_x_ concentration is relatively low, the concentration of NH_3_ being emitted through the stack is higher.

To verify this result, the NO_x_ data corresponding to the period of measurement at both power plants were obtained for comparison; the NO_x_ concentrations at power plants A and B were 7.62 and 4.43 ppm, respectively. The higher NH_3_ emission at power plant B is believed to be the result of the additional NH_3_ being used to reduce the level of NO_x_, allowing a higher concentration of NH_3_ to escape. This suggests that the related studies also showed the effects of NO_x_ reduction, and thus the relationship between NO_x_ and NH_3_ is inversely proportional [11,12,15]. Therefore, it is judged that the impact of the reduction of NO_x_ will be greater.

Currently in Korea, NH_3_ emission sources from LNG power plants are divided into two processes, energy fuel combustion and production, for the purpose of estimating the level of NH_3_ emissions. For the power plants utilized in this study, a separate SCR installation was not required and no additional processes following the SCR process caused exhaust gas emission through the stack, which led to the hypothesis that there would be no significant differences. To verify this, the mean distribution of the NH_3_ concentration based on the SCR and stack measurements at power plant A were compared by statistical analysis utilizing the SPSS 21(IBM) software. In general, when the number of samples is small, a nonparametric analysis can be carried out [16,17]. Therefore, due to the small number of NH_3_ samples at the SCR outlet and stack of power plant A, this study performed the comparison through mean correspondence utilizing the Wilcoxon signed-rank test, which is used for nonparametric distributions. The Wilcoxon signed-rank test compares the sum of the higher ranks, the median, and the sum of the lower ranks, after subtracting the median from the sample data and converting the resulting values into rank data. Therefore, the test takes into account sample data that are higher or lower than the median and the relative data size [18,19,20].

The results of the Wilcoxon signed-rank test, presented in Table 3, show that the level of significance was greater than 0.05, which indicates no significant difference in the median range of the NH_3_ concentration between the SCR outlet and the stack of both power plants, preserving the null hypothesis. Based on these results, it is not necessary to estimate the NH_3_ emission levels at LNG power plants separately for the energy fuel combustion and industrial process sectors.

### 3.2. NH_3_ Emission Factor and Comparison of NH_3_ Emissions

For this study, a total of 21 NH_3_ samples were collected at the stacks of power plants A and B, and an NH_3_ emission factor was calculated for the power plants. An NH_3_ emission factor was also calculated for the SCR outlet at power plant A. The emission factor results are presented in Table 4.

The results showed that the NH_3_ emission factor for the power plant stacks was 0.0054 ton NH_3_/m^3^, which is approximately nine times less than the U.S. EPA NH_3_ emission factor of 0.051 ton NH_3_/m^3^, currently applied in national statistics in Korea for LNG fuels in the industrial energy combustion sector. The SCR emission factor for power plant A was 0.0010 ton NH_3_/m^3^, which is considerably lower than 0.146 ton NH_3_/m^3^, the U.S. EPA NH_3_ emission factor currently applied in national statistics in Korea for LNG fuels in the industrial process sector. The results indicated substantial differences from the U.S. EPA emission factors currently applied in national statistics in Korea; therefore, an NH_3_ emission factor that incorporates Korean characteristics should be developed.

**Figure 4:**
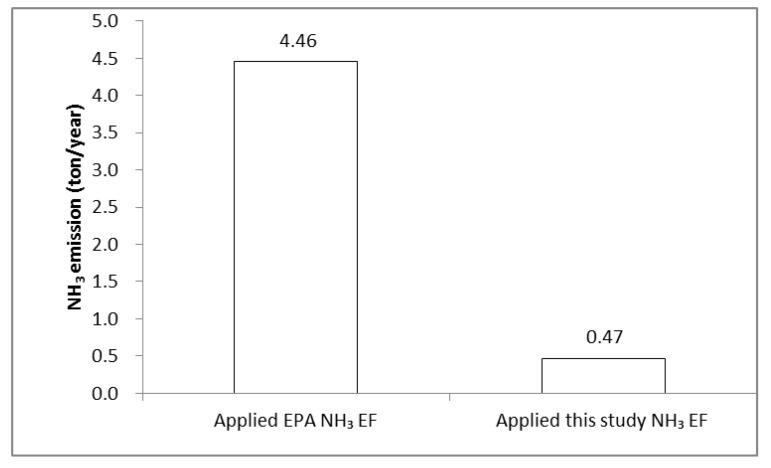
The comparison of NH_3_ EF (Emission Factor) for NH_3_ emissions at LNG power plants.

The emission factor for the LNG stack developed in this study and the EPA emission factor applied in conventional statistics in Korea were applied (fuel consumption of Korean LNG power plants in 2016: 87,395,623 Nm^3^/year), and the differences in the NH_3_ emission levels for LNG power plants were compared. The results of this comparison are presented in Figure 4.

The NH_3_ emission level estimated by applying the emission factor developed in this study was 0.47 ton NH_3_/year, a difference of approximately 3.99 ton from the NH_3_ emission level estimated by applying the conventional EPA emission factor of 4.46 ton NH_3_/year. Thus, NH_3_ emission factors reflecting the characteristics in Korea should be developed in order to improve the reliability of the inventory

### 3.3. Uncertainty of NH_3_ Emission Factor

**Figure 5:**
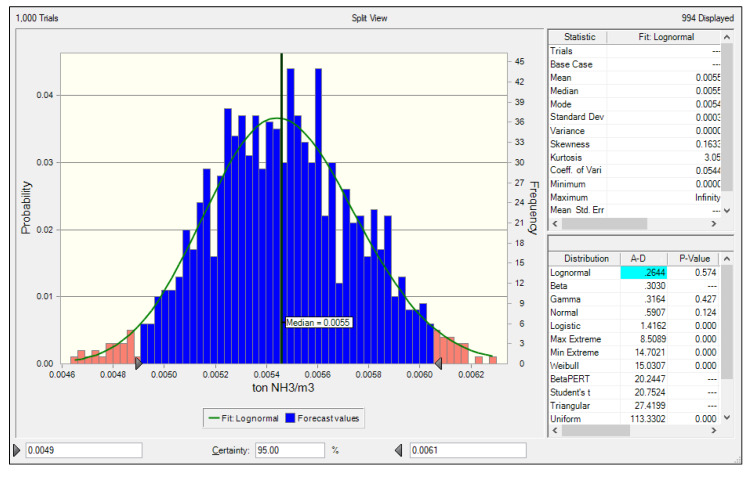
Uncertainty of the NH_3_ emission factor at the LNG stack.

**Figure 6:**
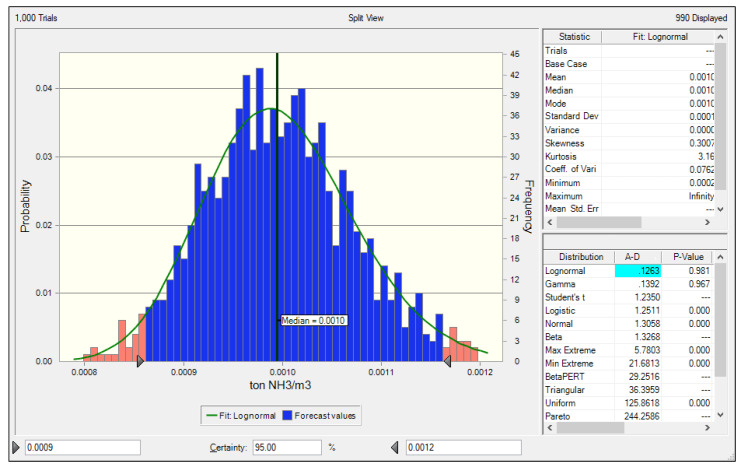
Uncertainty of the NH_3_ emission factor at LNG selective catalytic reduction (SCR) outlet.

The Monte Carlo simulation was used to estimate the uncertainty of the NH_3_ emission factor for LNG power plants developed in this study, and the results are presented in Figure 5 and Figure 6. The PDF of the NH_3_ emission factor for the LNG power plant stack developed in this study indicated a lognormal distribution. The median was 0.0055 ton NH_3_/10^6^Nm^3^ at a 95% confidence level, the lower 2.5% was 0.0049 ton NH_3_/10^6^Nm^3^, and the upper 97.5% was 0.0061 ton NH_3_/10^6^Nm^3^. Using these values, the estimated uncertainty range of the NH_3_ emission factor was ±10.91% at a 95% confidence level.

The PDF of the NH_3_ emission factor for the SCR outlet of the LNG power plant also indicated a lognormal distribution. The median was 0.0010 ton NH_3_/10^6^Nm^3^ at a 95% confidence level, the lower 2.5% was 0.0009 ton NH_3_/10^6^Nm^3^, and the upper 97.5% was 0.0012 ton NH_3_/10^6^Nm^3^. Using these values, the estimated uncertainty range of the NH_3_ emission factor was –10% to +20% at a 95% confidence level, which is a slightly higher range than that of the LNG stack.

Currently, the NH_3_ uncertainty range and numerical values are not available, which makes case comparison difficult. In Korea, the uncertainty of air pollutants is evaluated by the DARS (Data Attribute Rating System). Although the data rating system suggests several methods for converting various characteristics of the inventory into scores, such scores are based on the decision of experts and consequently dependent on subjective assessments, which poses limitations to the application of such scores as uncertainty values in practice [21]. Consequently, a quantitative assessment would be possible if the uncertainty range could be provided for air pollutants as it is for GHGs.

## 4. Conclusions

This study developed the NH_3_ emission factor for LNG power facilities in Korea by analyzing the emission characteristics from two LNG power plants using methods such as uncertainty analysis and comparing the differences in NH_3_ emission levels between the developed emission factors, which reflect the characteristics in Korea, and the U.S. EPA values currently applied in Korea. The study also analyzed the potential inventory improvement based on the differences in NH_3_ emissions from different sources.

Analyzing the NH_3_ concentrations at the LNG power plants showed a mean of 0.05 and 0.18 ppm for power plants A and B, respectively, indicating a substantial difference in the NH_3_ concentration, which was caused by the influence of the NO_x_ concentration. In addition, the NH_3_ concentration at the SCR outlet of power plant A was 0.04 ppm, showing no significant difference from the 0.05 ppm concentration at the stack. Furthermore, the statistical comparison found no difference between the emission factor based on NH_3_ concentration at the SCR outlet versus the stack as the final outlet. Therefore, it is preferable to apply the NH_3_ emission factor at the stack.

The estimation showed that the NH_3_ emission factor for the LNG power plants was 0.0054 ton NH_3_/10^6^Nm^3^, which is approximately nine times less than the EPA NH_3_ emission factor of 0.051 ton NH_3_/10^6^Nm^3^ for LNG fuels of the industrial energy combustion sector currently applied in national statistics in Korea. The SCR emission factor for LNG power plants was 0.0010 ton NH_3_/10^6^Nm^3^, which is considerably lower than the EPA NH_3_ emission factor of 0.146 ton NH_3_/10^6^Nm^3^ currently applied in national statistics in Korea for the LNG fuels of the industrial process sector. Furthermore, comparing the NH_3_ emission levels after applying the NH_3_ emission factor developed in this study to the EPA NH_3_ emission factor showed a difference of 3.99 ton NH_3_/year. This indicated the need for developing an emission factor that incorporates the unique characteristics in Korea.

The uncertainty range of the LNG stack NH_3_ emission factor developed in this study was ±10.91% at a 95% confidence level, while that of the SCR NH_3_ emission factor was –10% to +20% at a 95% confidence level, indicating a slightly higher uncertainty range than the LNG stack. At present, quantitative analysis of air pollutants is difficult because numerical values of the uncertainty are not available. However, quantitative analysis might be possible using the methods applied in this study to estimate uncertainty.

Two power plants were utilized in this study to investigate the NH_3_ emission factor and characteristics. This study’s significance mentioned the necessity of developing an NH_3_ emission factor that reflects the national characteristics by showing the difference and related characteristics between the NH_3_ emission factor and the measurement-based emission factor related to LNG plants currently applied in Korea. In addition, there are not many studies related to the NH_3_ emission from power plants, so it makes sense that a value that can be actually referenced was presented while presenting the relevant concentration range. However, it was not able to proceed due to certain consultation limitations with the power plant, such as seasonal effects and plant size-specific effects. In the future, if research is conducted on more LNG plants under smooth consultation, an NH_3_ emission factor that reflects Korea’s characteristics will be developed, and it will also help improve NH_3_ emission inventory reliability.

## Figures and Tables

**Table 1:** Characteristics of the investigated bituminous coal power plant.

Site	Capacity (MW)	Boiler Type	Fuel Type	Fuel Consumption (Nm^3^/day)	Flow Rate (Sm^3^/day)	Sampling Spot	Sampling
Power Plant A	450	Combined Cycle	LNG	1197,165	17,936,841	Stack	7
						SCR out let	7
Power Plant B	417	Combined Cycle	LNG	572,323	16,947,835	Stack	14

**Table 2:** NH_3_ concentration of the investigated bituminous coal power plant.

Site	Sampling Spot	NH_3_ Concentration (ppm)	NOx Concentration (ppm)	Sampling
LNG Power Plant A	Stack	0.05	7.23	7
		0.05	7.98	
		0.04	8.26	
		0.02	8.12	
		0.01	6.9	
		0.07	7.44	
		0.11	7.43	
	SD (standard deviation)	0.03	0.50	-
	Mean	0.05	7.62	-
	SCR outlet	0.01	7.23	7
		0.02	7.98	
		0.06	8.26	
		0.08	8.12	
		0.06	6.9	
		0.04	7.44	
		0.03	7.43	
	SD (standard deviation)	0.02	0.50	-
	Mean	0.04	7.62	-
LNG Power Plant B	Stack	0.04	4.28	14
		0.49	4.12	
		1.24	4.35	
		1.43	4.35	
		1.27	4.55	
		0.03	4.47	
		0.03	4.6	
		0.03	4.43	
		0.45	4.58	
		0.81	4.37	
		0.84	4.43	
		0.5	4.46	
		1.03	4.63	
		0.89	4.36	
	SD (standard deviation)	0.50	0.14	-
	Mean	0.65	4.43	-

**Table 3:** The result of Wilcoxon Singed Rank Test by NH_3_ concentration at LNG power plant for emission sources.

Hypothesis Test	Null Hypothesis	Test	Sig.	Decision
NH_3_ emission concentration at LNG power plant for emission sources	The median of differences between NH_3_ concentration of SCR and NH_3_ concentration of Stack equals 0	Related-samples Wilcoxon Singed Rank Test	0.735	Retain the null hypothesis

**Table 4:** NH_3_ emission factor of the investigated LNG power plant.

Classification	This Study (tonNH_3_/10^6^ Nm^3^)	US EPA(1994) (tonNH_3_/10^6^ Nm^3^)
LNG Stack	0.0054	0.051
LNG SCR	0.0010	0.146
